# Fatigue and its relationship with disease-related factors in patients with systemic sclerosis: A cross-sectional study

**DOI:** 10.3906/sag-2005-314

**Published:** 2021-04-30

**Authors:** Hazal YAKUT, Sevgi ÖZALEVLİ, Ahmet Merih BİRLİK

**Affiliations:** 1 Department of Institute of Health Sciences, Dokuz Eylül University, İzmir Turkey; 2 Department of School of Physical Therapy and Rehabilitation, Institute of Health Sciences, Dokuz Eylül University, İzmir Turkey; 3 Department of Immunology and Rheumatology, Faculty of Medicine, Dokuz Eylül University, İzmir Turkey

**Keywords:** Systemic sclerosis, fatigue severity, pulmonary function, respiratory muscle strength, health-related quality of life

## Abstract

**Background/aim:**

Fatigue is very common symptom in patients with systemic sclerosis (SSc) and adversely affects health-related quality of life and the ability to perform daily living activities. This study aimed to determine the severity of fatigue, and its related factors, in patients with SSc.

**Materials and methods:**

A total of 35 patients with SSc (6 males and 29 females, mean age of 50.71 ± 10.09 years) and 35 healthy control subjects (8 males and 27 females, mean age of 54.14 ± 9.51 years) were included in this study. The Fatigue Impact Scale for fatigue, Modified Medical Research Council Scale for dyspnea severity, 6-Minute Walking Test for functional capacity, Health Assessment Questionnaire Disability Index, Scleroderma Health Assessment Questionnaire and Short Form-36 Quality of Life Questionnaire for health-related quality of life were used in the evaluation of the subjects. Furthermore, pulmonary functions, diffusion capacity, and respiratory and peripheral muscle strength were evaluated.

**Results:**

Of the SSc patients, 80% experienced fatigue and presented with higher total and cognitive, physical, and psychosocial subscale fatigue scores than the healthy control subjects (P < 0.05). Moreover, the SSc patients exhibited significantly increased dyspnea severity, impaired pulmonary function-diffusion capacity, decreased respiratory-peripheral muscle strength, reduced functional capacity, and worsened health-related quality of life when compared to the control group (P < 0.05). Fatigue in the SSc group was significantly associated with age, dyspnea severity, diffusion capacity, respiratory and peripheral muscle strength, functional capacity, and health-related quality of life (P < 0.05).

**Conclusions:**

Along with the decrease in diffusion capacity, increase dyspnea, a decrease in both peripheral and respiratory muscle strength, and worsening functional capacity may have an effect on increased fatigue in SSc patients. Increased fatigue can also affect the life quality and daily life activities of a patient. Therefore, multidisciplinary approaches are recommended to evaluate and improve these parameters in the treatment of fatigue from the early period in SSc patients.

## 1. Introduction

Systemic sclerosis (SSc) is a multisystem disease that affects skin and internal organs via a disturbance in fibroblast function, vascular structure, and immune cell activation, resulting in fibrosis [1]. Due to this fibrosis, multiple organ and systemic involvement, such as pulmonary hypertension (PH), interstitial lung disease (ILD), skin thickening, finger ulcers, joint abnormalities and contractures, and kidney failure are very common. This multisystem involvement causes worsening prognosis, increased morbidity, and mortality in patients with SSc. Moreover, SSc is more prevalent in early-middle age and female sex [2,3].

Various factors, such as cardiopulmonary involvement, osteoarticular abnormalities, and musculoskeletal disorders associated with multisystem involvement, in SSc may induce fatigue, pain, decreased functional capacity, muscle strength loss, and respiratory symptoms [4]. However, general fatigue adversely affects health-related quality of life and the ability to perform daily living activities more than other symptoms. It was reported that the level of fatigue in these patients was as high as that of cancer patients reported in active therapy. Currently, etiological fatigue factors and its possible mechanism in SSc patients have not been fully elucidated, but it has been stated that skin tightening, system involvement, symptoms associated with comorbid conditions, inflammatory muscle disease, side effects of drug therapy, lifestyle, and psychosocial factors can cause fatigue [5]. 

Fatigue is a multidimensional symptom that is affected by many psychosocial, physical, and social factors. Moreover, it has been stated that fatigue is a major symptom in many inflammatory chronic rheumatoid diseases, including rheumatoid arthritis (80%–93%), ankylosing spondylitis (65%), and systemic lupus erythematous (81%), and has been associated with pain, female sex, longer disease duration, increased functional limitations, and depressive symptoms [6–8]. However, the number of clinical studies that have thoroughly investigated the frequency, features, and correlations of fatigue in SSc patients is insufficient. In addition, the evaluation of fatigue in the clinic is often neglected, although it has been stated that fatigue may be a significant source of discomfort and disability for SSc patients [9,10]. 

It is believed that studies examining the severity of fatigue and related factors in SSc are very important because they can provide evidence-based and better-targeted interventions during the follow-up period and in the management of the disease for these patients. Therefore, the first aim of this study was to determine the severity of fatigue, and its related factors, in patients with SSc. The second goal was to characterize fatigue severity, dyspnea severity, pulmonary functions, diffusion capacity, respiratory-peripheral muscle strength, functional capacity, and health-related quality of life of the patients to compare with a sample of healthy control subjects.

## 2. Patients and methods

### 2.1. Participants 

The study included 2 groups of volunteers, comprising the SSc group and healthy control group, with 35 participants in each group. Patients with SSc were diagnosed according to the American College of Rheumatology/European League Against Rheumatism criteria by a rheumatologist and followed-up at the Department of Immunology-Rheumatology, between 2017 and 2019 [11]. The inclusion criteria were being between the ages of 35–65 years and volunteering to participate to the study. The exclusion criteria were having other diseases that may affect the results (mental, neurological, musculoskeletal, cardiopulmonary disease not related to SSc, acute infection) and difficulty in walking. The patients were stable in terms of their medical treatment and were asked to continue their medical treatment regularly throughout the study. 

The control group consisted of healthy volunteers who were between the ages of 35–65 years who had been recruited from the Dokuz Eylül University, Faculty of Medicine employees. These participants did not show evidence of any disorders and/or diseases that may have affected the results (mental, neurological, cardiovascular, pulmonary, musculoskeletal disease).

### 2.2. Assessment

The study design was cross-sectional. Age, weight, height, body mass index (BMI), disease duration (in years), respiratory symptoms (cough, sputum, and dyspnea, etc.), and other involvement were recorded. Fatigue and dyspnea severity, pulmonary function, diffusion capacity, respiratory and peripheral muscle strength, functional capacity, and health-related quality of life of the individuals were evaluated, respectively. Furthermore, between the tests, the subjects rested for 10 min to avoid test-related fatigue. 

#### 2.2.1. Fatigue impact scale

The Fatigue Impact Scale (FIS) measures physical, cognitive, and social influences of fatigue. It consists of 40 items and a high score indicates a high level of fatigue. Furthermore, it consists of 3 subscales: cognitive, physical, and psychosocial [12]. 

#### 2.2.2. Modified Medical Research Council Scale

The dyspnea severity of the patients was evaluated using the Modified Medical Research Council Scale (mMRCS). The mMRCS questions the dyspnea severity in daily living activities. The mMRCS contains 5 degrees (range 0–4) and a higher score indicates a higher perceived amount of breathing [13]. 

#### 2.2.3. Pulmonary function and diffusion capacity

The pulmonary function test was applied using a Sensor Medics Vmax 22 0.6-2B version spirometer (ERS 1993 Uptake + Zapleta, SensorMedics, Inc, Anaheim, CA, USA). Forced vital capacity (FVC), forced expiratory volume in 1 s (FEV1), FEV1/FVC ratio, and peak expiratory flow (PEF) values were recorded according to the American Thoracic Society/European Respiratory Society criteria [14]. The single breath carbon monoxide method was used to determine the diffusion capacity for carbon monoxide (DLCO) measurement [15].

#### 2.2.4. Respiratory and peripheral muscle strength

An electronic mouth pressure measuring device was used to determine the respiratory muscle strength. The maximal inspiratory pressure (MIP) and maximal expiratory pressure (MEP) were measured. The MIP was measured as the residual volume upon a maximal inspiratory effort against an occluded airway and the MEP was measured as the total lung capacity while performing a maximal expiratory effort against an occluded airway. The MIP and MEP measurements were repeated 3 times, and the maximum value achieved was recorded [16]. In order to evaluate the muscle strength of the lower extremities, the dominant quadriceps muscle group was determined and the muscle strength was measured. The quadriceps muscle function test included an isometric maximal voluntary contraction force test that was performed using a Hoggan Scientific MicroFET2 Digital Dynamometer & Manual Muscle Tester (Salt Lake City, UT, USA). Test was performed with the patient in a sitting position with 90° hip flexion and 60° knee extension over the end of a chair. The patients were asked to continue the maximum isometric contraction for 5 s and the average of the measured values was obtained by performing 3 successive maximal contractions. Handgrip strength was measured to roughly define the upper extremity muscle strength. The measurement was performed using a Jamar Hydraulic Hand Dynamometer (Patterson Medical Products, Warrenville, IL, USA ) in a sitting position, with shoulder adduction and neutral rotation, elbow at 90° flexion, forearm in mid rotation and supported, and wrist in the neutral position. For the dominant hand, 3 measurements were made by giving 1-min intervals between each measurement, and the mean was recorded [17,18].

#### 2.2.5. Functional capacity

The functional capacity of the patients was evaluated using the 6-Minute Walking Test (6MWT). For the 6MWT, all of the patients were asked to walk as fast as possible for 6 min down a flat, 30-m-long corridor. The total distance, in meters, was recorded. The predicted walking distance value for each patient was calculated by means of the equations formulated by Enright et al., as recommended by the American Thoracic Society. For the safety of the patients, before and after the tests, their heart rates, blood pressure, and oxygen saturation were checked [19]. 

#### 2.2.6. Health-related quality of life

The Health Assessment Questionnaire Disability Index (HAQ-DI), Scleroderma Health Assessment Questionnaire (SHAQ), and Short Form-36 Quality of Life Questionnaire (SF-36) were used for assessment of health-related quality of life.

The HAQ-DI measures disability, function, and quality of life using 8 functional domains of physical capacity (dressing and grooming, arising, eating, walking, hygiene, reach, grip, and common daily activities), which consist of 20 items. Each item is scored between 0 (without any difficulty) and 3 (unable to do). The SHAQ also has a visual analogue scale (VAS) of 5 SSc symptoms and signs (Raynaud’s phenomenon, digital ulcers, gastrointestinal problems, pulmonary problems, and global assessment of the severity of the disease). The SHAQ-global score was calculated by adding the 5 SSc-related VAS symptoms to the 8 HAQ-DI domains and dividing the sum by 13 [20,21]. The SF-36 measures health-related quality of life and consists of 36 items in total, and includes 2 subscales, which are physical and mental. The subscales are scored between 0 and 100 and a low score indicates poor health status [22].

### 2.3. Statistical analysis

All of the data were analyzed using IBM SPSS Statistics for Windows 22.0 (IBM Corp., Armonk, NY, USA). The normal distribution of the data was examined using the Shapiro-Wilk test. Descriptive statistics were presented as frequency and percentage (%) for the categorical variables and the mean ± standard deviation according to the distribution of the continuous variables. The independent-samples t test and chi square test (percentages, categorical variables) were used for comparisons between 2 groups. The correlation between the studied variables was evaluated using the Pearson correlation coefficient. A type I error level of 5% was accepted as statistically significant. To obtain a power of 85% with a probability of a 2-tailed type I error of 0.05, a sample of 35 participants was required in each group to demonstrate a significant difference on the FIS score [23]. Power analysis was calculated using G*Power 3.1 (Heinrich Heine University Düsseldorf, Düsseldorf, Northrhine-Westphalia, Germany) software.

## 3. Results

Participating in this study were 35 patients with SSc (6 males and 29 females, mean age of 50.71 ± 10.09 years) and 35 healthy subjects (8 males and 27 females, mean age of 54.14 ± 9.51 years). The mean disease duration was 7.64 ± 5.38 years. The demographic and clinical characteristics of the subjects are listed in Table 1. The healthy control subjects were well-matched with the SSc group for age, sex, and BMI (P > 0.05). The subjects who quit smoking at least 9 months prior to the start of the study were included, and there was no difference between the 2 groups in terms of the number of former smokers and smoking history (P > 0.05). Moreover, the participants did not have regular exercise habits. Compared with the healthy control group, the SSc group showed significantly higher pulmonary symptoms (cough and sputum) and dyspnea severity (P < 0.05). Of the patients, 20 (57.1%) had diffuse SSc and 15 (42.9%) had limited disease. Arthralgia and complaints compatible with gastrointestinal disease were reported by 14 (40.0%) and 10 (28.57%) of the patients with SSc, respectively. Of the patients, 28% (80.0%) had Raynaud’s syndrome and 5 (14.28%) had digital ulcerations. 

**Table 1:** Demographic and clinical characteristics of the patients with SSc and the healthy subjects.

	SSc patients(n = 35)	Healthy individuals(n = 35)	P-value
Sex (male/female), n	6/29	8 / 27	0.550
Age (years)	50.71 ± 10.09	54.14 ± 9.51	0.369
Disease duration (years)	7.64 ± 5.38	-	-
Height (cm)	162.02 ± 7.55	166.34 ± 6.56	0.401
Weight (kg)	70.59 ± 15.59	76.42 ± 13.56	0.451
BMI (kg/m2)	26.10 ± 4.76	27.29 ± 3.84	0.599
Former smoker, n (%)	7 (20.0)	8 (22.8)	0.771
Smoking history (packet-years)	2.4 ± 6.09	2.25 ± 5.33	0.743
Type of disease			
Diffuse SSc, n (%)	20 (57.1)	-	-
Limited SSc, n (%)	15 (42.9)	-	-
PH n (%)	14 (40.0)	-	-
ILD n (%)	18 (51.4)	-	-
Pulmonary symptom			
Cough, n (%)	17 (48.5)	1 (2.8)	< 0.001
Sputum, n (%)	13 (37.1)	1 (12.8)	0.001
Dyspnea severity (mMRCS score)	2.60 ± 0.69	0.14 ± 0.35	< 0.001
Gastrointestinal symptom	10 (28.57)	-	-
Arthralgia	14 (40.0)	-	-
Digital ulcerations, n (%)	5 (14.28)	-	-
Raynaud’s syndrome, n (%)	28 (80.0)	-	-

Data are presented as the number, percentage, and/or the mean ± standard deviation; BMI: body mass index; PH: pulmonary hypertension; ILD: interstitial lung disease; mMRCS: modified medical research council scale.

The comparisons of the total and cognitive, physical, and psychosocial subscale fatigue scores, pulmonary function test results, diffusion capacity, and respiratory muscle strength values of the between the SSc group and healthy control group are shown in Table 2. The SSc patients had significantly lower total and cognitive, physical, and psychosocial subscale fatigue scores than the controls (P < 0.001). Patients with SSc showed lower FEV1%, FVC%, PEF%, DLCO%, MIP%, and MEP% (P < 0.05) than the controls, whereas there was no difference in the FEV1/FVC% between the groups (P > 0.05).

**Table 2:** Comparison of fatigue severity and pulmonary function, diffusion capacity, and respiratory muscle strength in the patients with SSc and the healthy subjects.

	SScpatients (n = 35)	Healthy individuals(n = 35)	P-value
Fatigue severity n (%)	28 (80.0)	6 (17.1)	<0.001
FIS total score	74.62 ± 31.54	11.05 ± 10.20	<0.001
FIS cognitive score	15.37 ± 7.84	5.65 ± 4.13	<0.001
FIS physical score	23.48 ± 8.36	4.37 ± 3.88	<0.001
FIS psychosocial score	35.77 ± 17.22	6.02 ± 4.55	<0.001
Pulmonary function test			
FVC (%pred)	76.82 ± 18.24	94.62 ± 6.86	<0.001
FEV1 (%pred)	78.08 ± 19.78	93.06 ± 5.15	<0.001
FEV1/FVC (%pred)	103.45 ± 8.39	95.34 ± 5.24	0.035
PEF (%pred)	74.57 ± 22.26	92.68 ± 11.17	<0.001
DLCO (%pred)	61.88 ± 17.25	95.40 ± 6.73	<0.001
MIP cmH2O (%pred)	58.54 ± 31.33	99.03 ± 17.23	0.001
MEP cmH2O (%pred)	57.80 ± 23.18	116.42 ± 15.17	0.022

Data are presented as the number, percentage, and/or the mean ± standard deviation; FIS: Fatigue Impact Scale; FVC: forced vital capacity; FEV1: forced expiratory volume in 1 second; PEF: peak expiratory flow; DLCO: diffusion capacity for carbon monoxide; MIP: maximal inspiratory pressure; MEP: maximal expiratory pressure, %pred: percent predicted.

 The comparisons of the peripheral muscle strength, functional capacity, and health-related quality of life results of between the SSc group and healthy control group are shown in Table 3. In addition, the results of the subparameters of the HAQ-DI and SHAQ, evaluating the disease-specific quality of life, are also indicated in Table 3 for the SSc group. The SSc patients exhibited significantly weaker peripheral muscle strength and shorter walking distance than the healthy controls (P < 0.05). The SSc patients had significantly lower physical and mental category scores on the SF-36 than the healthy controls (P < 0.05).

**Table 3:** Comparison of the peripheral muscle strength, functional capacity, and health-related quality of life results of the patients with SSc and the healthy subjects.

	SSc patients (n = 35)	Healthy individuals(n = 35)	P-value
Peripheral muscle strength			
QF strength (kg)	6.84 ± 1.84	17.40 ± 5.46	<0.001
Handgrip (kg)	16.47 ± 3.77	36.35 ± 5.23	0.034
Functional capacity			
6MWT walking distance (m)	395.08 ± 75.00	546.34 ± 42.30	0.035
6MWT walking distance (%pred)	66.0 3 ± 10.85	98.07 ± 4.96	0.026
Health-related quality of life			
HAQ-DI	1.06 ± 0.60	-	-
SHAQ-Raynaud’s phenomenon VAS	1.32 ± 0.51	-	-
SHAQ-digital ulcer VAS	0.84 ± 0.49	-	-
SHAQ-digestive VAS	0.92 ± 0.45	-	-
SHAQ-pulmonary VAS	1.35 ± 0.73	-	-
SHAQ-overall disease severity VAS	1.47 ± 0.67	-	-
SHAQ-global	1.12 ± 0.54	-	-
SF-36 physical score	39.60 ± 18.38	82.77 ± 6.79	<0.001
SF-36 mental score	42.57 ± 15.20	80.51 ± 5.60	<0.001

Data are presented as the mean ± standard deviation; QF: quadriceps femoris muscle; 6MWT: 6-Minute Walking Test; HAQ-DI: Health Assessment Questionnaire Disability Index; SHAQ: Scleroderma Health Assessment Questionnaire; VAS: visual analogue scale, SF-36: Short Form-36 Quality of Life Questionnaire.

Total fatigue in SSc group was significantly associated with age, dyspnea severity, pulmonary function, diffusion capacity, respiratory-peripheral muscle strength, functional capacity, and health-related quality of life (Table 4). Cognitive fatigue scores in the SSc group were significantly associated with age, dyspnea severity, handgrip and lower-limb muscle strength, functional capacity, and health-related quality of life (SF-36, HAQ-DI, and SHAQ) (P < 0.05). Physical fatigue scores in the SSc group were significantly associated with dyspnea severity, pulmonary function (FEV1%, FVC%, PEF%), DLCO, MIP, MEP, handgrip and lower-limb muscle strength, functional capacity, and health-related quality of life (SF-36, HAQ-DI, and SHAQ) (P < 0.05). Psychosocial fatigue scores in the SSc group were significantly associated with dyspnea severity, MEP, handgrip and lower-limb muscle strength, functional capacity, and health-related quality of life (SF-36, HAQ-DI, and SHAQ) (P < 0.05). 

**Table 4:** Correlations between the fatigue severity and demographic data, dyspnea severity, pulmonary function, peripheral muscle strength, functional capacity, functional disability, and health-related quality of life of the SSc patients.

	FIStotal	FIScognitive	FISphysical	FISpsychosocial
				
Age (years)	0.337*	0.355*	0.278	0.320
Disease duration (years)	0.071	0.152	0.004	0.059
BMI (kg/m2)	0.063	-0.082	0.141	0.084
Dyspnea severity (mMRCS score)	0.621**	0.595**	0.612**	0.570**
FVC (%pred)	–0.276	–0.238	–0.359*	–0.237
FEV1 (%pred)	–0.303	–0.236	–0.386*	–0.274
FEV1 / FVC (%pred)	0.056	–0.195	–0.047	–0.009
PEF (%pred)	–0.319	–0.203	–0.394*	–0.300
DLCO (%pred)	–0.352*	–0.321	–0.359*	–0.326
MIP (%pred)	–0.358*	–0.300	–.0385*	–0.332
MEP (%pred)	–0.375*	–0.324	–0.387*	–0.387*
Quadriceps strength (kg)	–0.407*	–0.372*	–0.400*	–0.382*
Handgrip strength (kg)	–0.403*	–0.348*	–0.503**	–0.347*
6MWT walking distance (m)	–0.509**	–0.369*	–0.491**	–0.526**
6MWT walking distance (%pred)	–0.379*	–0.374*	–0.439**	–0.444**
HAQ-DI	0.751**	0.699**	0.729**	0.704**
SHAQ-global	0.784**	0.715**	0.752**	0.745**
SF-36 physical score	–0.776**	–0.616**	–0.692**	–0.687**
SF-36 mental score	–0.712**	–0.710**	–0.708**	–0.754**

*0.01< P ≤0.05, **P ≤ 0.01, r: Pearson’s correlation coefficient.

## 4. Discussion

According to the results of the current study, 80% of the SSc patients experienced fatigue, and the SSc patients had higher total and cognitive, physical, and psychosocial subscale scores of fatigue than the healthy controls. Fatigue in the SSc group was significantly associated with age, dyspnea severity, diffusion capacity, respiratory and peripheral muscle strength, functional capacity, and health-related quality of life. Many studies have reported that fatigue is very common in SSc patients, similar to the results herein [10,24]. Sandusky et al. [25] reported a prevalence of fatigue at 76% in SSc patients. Thus far, few studies have fatigue correlations and predictors in patients with SSc in a sufficiently broad and comprehensive manner. Willems et al. [26] reported that fatigue was associated with lung involvement, female sex, and low coping and acceptance skills in patients with SSc. In a study by Thombs et al. [5], it was stated that a higher number of comorbidities, respiratory problems, smoking, gastrointestinal symptoms, and depression were associated with higher levels of fatigue in patients with SSc. Unlike previous studies, in addition to the clinical features and symptoms of patients, it is our belief that the current study was more comprehensive, by examining the relationship between fatigue and dyspnea severity, respiratory-peripheral muscle strength, functional capacity, and health-related quality of life. 

Due to lung involvement, poor ventilation distribution, lung membrane thickening, and pulmonary capillary bed loss can potentially lead to structural changes in airways, resulting in a loss of air flow that can be reflected by increased ventilator demand in SSc patients [27]. Therefore, the use of a spirometer and DLCO measurement is very common and important in the diagnosis and follow-up of SSc patients [28]. Similar to other studies, lower pulmonary function, DLCO, and respiratory muscle strength was also observed in the patients with SSc when compared to the controls [5,29]. In the current study, fatigue showed a significant correlation with DLCO and respiratory muscle strength, but not with pulmonary functions. Thus, decreased respiratory muscle strength and diffusion capacity increase fatigue severity, rather than impair pulmonary functions in this population. It is our belief that respiratory muscle strength measurement, similar to DLCO, reflects the clinic of patients more than the pulmonary function tests. Therefore, an evaluation of respiratory muscle strength is recommended from the early period in the diagnosis and follow-up of patients with SSc. Moreover, when the subscales of fatigue are examined, it can be seen that decreased pulmonary function, DLCO, and respiratory muscle strength affected physical fatigue more than cognitive and psychosocial fatigue.

It has been reported that skin-tightening limiting movement and chest expansion, ILD, and PH may cause dyspnea in SSc patients [5]. No comprehensive studies that examined the relationship between fatigue and dyspnea severity in SSc patients could be found in the literature. However, the dyspnea severity, which was significantly higher in the SSc patients than in the healthy controls in the current study, was correlated with the total and cognitive, physical, and psychosocial subscale fatigue scores. As lung involvement increases in the patients, the dyspnea severity may increase, which may, in turn, limit the functionality of the patients. Therefore, it is our belief that this indirectly increased dyspnea severity may affect the level of fatigue. For this reason, it should not be ignored that an assessment of dyspnea, due to many different reasons, may occur in this patient group and dyspnea severity reduction strategies should be suggested in fatigue management.

Skeletal muscle involvement in SSc ranges from 24% to 97%. Muscle involvement in the form of myositis or noninflammatory myopathy in SSc patients causes general weakness and muscle atrophy [30]. Hand grip strength can be generalized as a global muscle strength marker in SSc. However, strength loss rates differ in the upper and lower extremities, with a greater decrease in muscle strength in the lower extremities than in the upper extremities [31]. Therefore, an evaluation of both upper- and lower-limb strength is important to reveal true peripheral muscle weakness. The upper and lower-limb muscle strength of the patients in the current study was lower than that in the healthy controls, which was similar to the literature [23,29]. Contrary to the correlation between fatigue and upper-lower muscle strength, which was observed in the current study, no studies that investigated the relationship between general fatigue severity and peripheral muscle strength could be found in the literature. In addition to the upper-limb, the lower-limb muscle strength, which is one of the determinants of functional capacity, decreases in these patients and affects the severity of fatigue. This result suggested that weakness and atrophy in both upper- and lower-limb muscles may increase fatigue more quickly, and approaches to maintain and increase peripheral muscle strength can be effective in managing fatigue.

Similar to the findings herein, it was reported in other studies that walking distance and its predicted value decreased in SSc patients when compared to healthy controls [4,19,29]. Changes in the small blood vessels of the skeletal muscles due to vascular damage may also have a negative effect on the oxygen supplied to the cells, which contributes to weaker exercise performance and functional capacity [32]. Deuschle et al. [4] investigated the 6MWT and associated clinical parameters in patients with SSc and found that the 6MWT was correlated with disease activity, SHAQ score, nutrition status, age, hemoglobin values, and several lung function parameters, but did not investigate its relationship with fatigue. In the current study, in addition to being related to total fatigue and functional capacity in the SSc patients, the subscales of the FIS were also evaluated, and it was determined that fatigue affected both the cognitive and psychosocial states, as well as the physical state, and they were correlated with functional capacity. These results showed that individuals with more social and cognitive fatigue may have lower functional capacity, due to lower motivation and social involvement.

The patient sample herein had a mean HAQ-DI score of ≥1.0, which was similar to that in other studies, and this cut-off was important because it was associated with high morbidity and mortality in this patient group over a period of 4 years [33–35]. Also observed in the current study was a correlation between the HAQ-DI and the total and cognitive, physical, and psychosocial subscale fatigue scores. Many studies have confirmed that fatigue is one of the most important symptoms in SSc, which has a significantly negative impact on the functional disability, living, and quality of life of patients [35,36]. The mean SHAQ score in the patient group was similar to that of other studies, while the SF-36 physical and mental scores were significantly lower than those in the healthy group [29,37,38]. In a study conducted by McNearney et al. [38], fatigue was correlated with the physical component of the SF-36 in SSc patients. Jacoub et al. [37] demonstrated a strong association between fatigue and health-related quality of life (all domains of the SF-36 and SHAQ) in SSc patients, as in the current study. Fatigue severity increases with a decrease in pulmonary functions and diffusion capacities, increase in dyspnea severity, and decrease in respiratory-peripheral muscle strength and functional capacities. As a result of this, the daily life activities and participation of patients are limited, and their quality of life deteriorates. Therefore, adequate fatigue management and related factors can lead to improvements in functional disability, and ultimately, to a significant improvement in the quality of life of these patients.

The strongest aspect of this study was that it was the first comprehensive study to evaluate the total, and cognitive, physical, and psychosocial aspects of fatigue, and examine their relationship with disease-related parameters in SSc patients. This study had several limitations. First, the influences of additional factors, possibly related to fatigue in SSc patients, were not evaluated, such as inflammatory cytokines, serum markers, and medication. Second, this study was cross-sectional and in the study sample, the results were not separated according to the type of disease (diffuse or limited). Therefore, further research is suggested to determine the effects of these additional factors on fatigue in different types of SSc.

The results showed that SSc patients experienced more frequent and severe fatigue when compared to the healthy controls. Moreover, increased fatigue severity was significantly associated with the age, dyspnea severity, pulmonary function, diffusion capacity, respiratory and peripheral muscle strength, functional capacity, and health-related quality of life in the SSc population herein. This suggested that fatigue severity in patients with SSc is not solely associated with clinical features and symptoms. Therefore, fatigue severity and these disease-related factors should be evaluated comprehensively in the clinic. Moreover, in SSc patients, respiratory exercises and respiratory muscle training for increased ventilation and respiratory muscle strength; dyspnea reduction strategies for decreased dyspnea; aerobic and resistant exercise programs for increased peripheral muscle strength; daily life activity training, and energy conservation techniques can be recommended and applied from the early period in fatigue management. As a result, the quality of life of the patients and their participation in daily life activities can be increased through fatigue management. In addition, we believe that the results of this study support the need for further research on fatigue and its related factors in patients with SSc.

## Informed consent

Ethical approval was obtained from the Non-Invasive Research Ethics Committee of Dokuz Eylül University (No: 2017/29-15, Protocol Number: 3701-GOA, Date: 21.12.2017) prior to the study and all procedures were conducted in accordance with the Declaration of Helsinki. Signed informed consent forms were obtained from all of the participants prior to the study.

## Funding

The authors received no financial support for the research and/or authorship of this article.
